# Coronary artery calcium scoring: expanding the new standard by photon-counting detector CT Part II: Impact of virtual monoenergetic image reconstructions with adjusted calcium scoring thresholds

**DOI:** 10.1007/s00330-026-12356-3

**Published:** 2026-02-19

**Authors:** Nicola Fink, Lennart R. Koetzier, Emese Zsarnoczay, Milan Vecsey-Nagy, Dmitrij Kravchenko, Muhammad Taha Hagar, Jim O’Doherty, Moritz C. Halfmann, Pal Suranyi, Gijs D. van Praagh, Jens Ricke, Pal Maurovich-Horvat, Tobias Bäuerle, Martin J. Willemink, Akos Varga-Szemes, Tilman Emrich

**Affiliations:** 1 Department of Radiology and Radiological Science, Medical University of South Carolina, Charleston, SC USA; 2 Department of Radiology, University Hospital, LMU Munich, Munich, Germany; 3 Department of Radiology, Stanford University School of Medicine, Stanford, CA USA; 4 Department of Radiology, Medical Imaging Centre, Semmelweis University, Budapest, Hungary; 5 Heart and Vascular Center, Semmelweis University, Budapest, Hungary; 6 Department of Diagnostic and Interventional Radiology, University Hospital Bonn, Bonn, Germany; 7 Department of Diagnostic and Interventional Radiology, Medical Center–University of Freiburg, Faculty of Medicine, University of Freiburg, Freiburg, Germany; 8 Siemens Medical Solutions, Malvern, PA USA; 9 Department of Diagnostic and Interventional Radiology, University Medical Center of Johannes Gutenberg-University, Mainz, Germany; 10 German Centre for Cardiovascular Research, Partner site Rhine-Main, Mainz, Germany; 11 MTA-SE Cardiovascular Imaging Research Group, Department of Radiology, Medical Imaging Centre, Semmelweis University, Budapest, Hungary

**Keywords:** Coronary artery calcium, Photon-counting detector CT, Agatston score, Phantom study, Virtual monoenergetic images

## Abstract

**Objectives:**

Part I of this study introduced a new photon-counting detector (PCD-)CT protocol for coronary artery calcium (CAC) scoring using 120 kVp, 75% dose, thin slices, quantum iterative reconstructions (IR) 2, leading to a significant reduction of score variability. The second part evaluated the potential of virtual monoenergetic image (VMI) reconstruction in further reducing score variability with PCD-CT.

**Materials and methods:**

CAC scoring was performed on PCD-CT with a chest phantom containing nine calcifications using the optimized PCD-CT protocol from Part I. Images were reconstructed at different VMI levels (50–80 keV, 5 keV-steps), with adjusted CAC thresholds to maintain density equivalence to 70 keV. CAC scores, image noise, and calcification detectability were investigated. Results were compared to standard PCD-CT, EID-CT and previously proposed EID-CT protocols.

**Results:**

Using 65 keV reconstructions, score variability decreased by 9% compared to the optimized PCD-CT protocol from Part I, by 43% vs. the standard PCD-CT, by 78% vs. the standard EID-CT, and by 69% vs. the proposed EID-CT protocol. Image noise remained within targets, eliminating the risk of false-positives. Calcification detectability was comparable to the optimized PCD-CT protocol (7.1 ± 0.6 vs. 7.1 ± 0.8). Calcium volume and mass scores from the keV-optimized PCD protocol were closer to the physical reference compared to scores from the standard PCD protocol.

**Conclusions:**

Score variability and calcification detectability in PCD-CT-based CAC scoring can be further improved when augmenting an optimized PCD-CT protocol at 65 keV. In addition to reducing the radiation dose, this protocol may enable more consistent CAC quantification and seems to perform even better than the proposed, multivendor EID-CT protocol.

**Key Points:**

***Question*** *Coronary calcium scoring lacks reproducibility. Adding virtual monoenergetic imaging with adapted thresholds to a pre-optimized photon-counting CT protocol may further improve score variability.*

***Findings*** *A 120 kVp, 75%-dose, thin-slice photon-counting CT protocol at 65 keV achieved the lowest coronary calcium score variability compared to previous protocols.*

***Clinical relevance*** *Minimizing variability in coronary calcium scoring improves the technical reliability of serial measurements. The additional use of virtual monoenergetic imaging further reduces variability in photon-counting CT, supporting a precise and consistent cardiovascular risk assessment.*

**Graphical Abstract:**

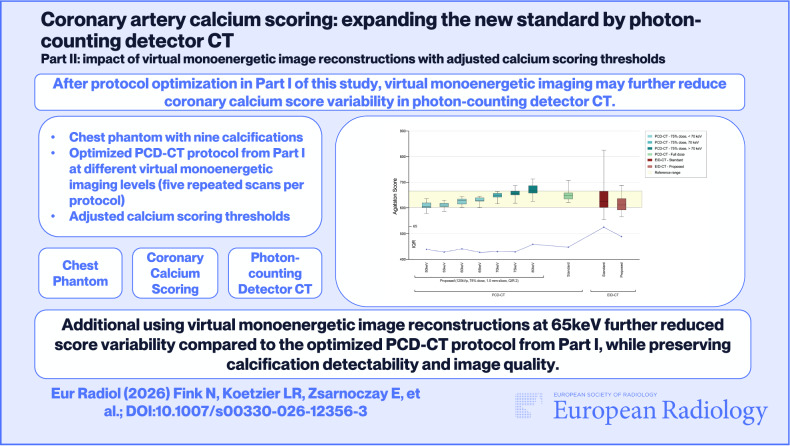

## Introduction

Accurate and reproducible quantification of coronary artery calcium (CAC) is essential for cardiovascular risk assessment and follow-up. Building on the optimization of CAC scoring parameters on photon-counting detector (PCD) CT in Part I of this study, the present investigation further explores technical parameters that are available with PCD-CT, aiming for an even more reliable CAC scoring with PCD-CT.

CAC scoring plays a pivotal role in assessing the risk of coronary artery disease [1, 2] and is used to improve clinical management of patients with borderline or intermediate cardiovascular risk [3]. Nevertheless, current CAC methods are known for their limited score reproducibility, which potentially leads to risk reclassification [4–6] and subsequent variations in treatment decisions. At the same time, variations in scan parameters and reconstruction settings have a notable impact on CAC quantification [7–13], emphasizing the need for standardized and optimized protocols for CAC assessment. For energy-integrating detector (EID) CT, van Praagh et al [7] proposed a multivendor-validated CAC protocol that reduced intra- and interscanner variability and improved calcification detectability.

Accordingly, in Part I of this study, we applied different acquisition and reconstruction parameters on CAC scoring with PCD-CT and analyzed their impact on score variability. Using a protocol at 120 kVp, 75% radiation dose, and reconstructed at thin slices with quantum iterative reconstructions (IR) strength level 2, led to a significant reduction of CAC score variability as well as improved calcification detectability on PCD-CT. In addition to these parameters, PCD-CT offers spectral capabilities, particularly the reconstruction of virtual monoenergetic images (VMI), which can be utilized to further optimize image quality and diagnostic accuracy [14, 15].

Therefore, Part II of this study aimed to evaluate the potential of VMI reconstructions to further improve CAC score variability with PCD-CT when augmenting the optimized protocol proposed in Part I. Results were compared with corresponding multivendor values from four state-of-the-art EID-CT systems.

## Materials and methods

In this prospective phantom study, no humans or animal subjects were involved. Therefore, institutional review board approval and informed consent were not required.

To maintain consistency and enable comparison, the study was structured in accordance with the methodology by van Praagh et al [7]. The identical phantom model was employed, and acquisition and reconstruction parameters were matched.

### Phantom

In this prospective phantom study, CAC scoring was performed using an anthropomorphic phantom (QRM) containing nine calcifications with different diameters (5, 3, and 1 mm), and calcium hydroxyapatite (CaHA) densities (800, 400, and 200 mg/cm^3^), along with two calibrations rods of known CaHA (200 mg/cm^3^) and water-equivalent densities. Using extension rings, the phantom simulated small (anterior-posterior × lateral: 30 × 20 cm), medium (35 × 25 cm) and large (40 × 30 cm) patients.

### Data acquisition and image reconstruction

PCD-CT (NAEOTOM Alpha; Siemens Healthineers) scans were performed using settings according to van Praagh et al [7], as described in Part I. To specifically assess interscan variability, each protocol was repeated five times with slight transitional (5 mm) and rotational (2°) phantom movements [16]. The same repositioning approach was applied as described in Part I to ensure methodological consistency across both parts of this study and comparability with the study by van Praagh et al [7]. Standard radiation dose (100%) was based on clinical protocols with volumetric CT dose indices of 1.5, 3.3, and 7.0 mGy for the small, medium, and large phantoms, respectively. A proprietary offline raw data reconstruction platform (ReconCT version 15.0.57554.0; Siemens) was used for reconstructions. As proposed in Part I of this investigation, a protocol acquired at 120 kVp, 75% dose with thin slices (1.0 mm thickness, 1.0 mm increment) and IR2 was selected for further analyses. Images were reconstructed at different VMI from 50–80 keV (5 keV-steps).

### Image and statistical analysis

A validated, fully automated quantification method was used for CAC quantification [7, 17]. Different VMI levels are known to influence CAC scoring [18]: Lower keV settings enhance calcifications, potentially increasing scores. The current standard CAC quantification is performed using 70 keV at a CAC threshold of 130 HU [19]. Due to the impact of different keV, CAC thresholds used for the Agatston scores have to be adjusted when using different VMI levels for CAC scoring and the calibration factor used for mass and volume scores. This was conducted based on previous studies, performing CAC threshold adaptation at various kVp [20, 21].

The Agatston score method depends on calcium attenuation [19]. To achieve equal physical density compared to the standard threshold (*t*) of 130 HU at 70 keV, thresholds were adapted using the following formula per VMI (*xkeV*) based on the attenuation of the CaHA calibration insert. Furthermore, weighting factors for Agatston score analysis were adapted.

$${{{{\rm{t}}}}}_{{{{\rm{xkeV}}}}}=130\,{{{\rm{HU}}}}\times \frac{{{{{\rm{HU}}}}}_{{{{\rm{CaHA@xkeV}}}}}}{{{{{\rm{HU}}}}}_{{{{\rm{CaHA@}}}}70{{{\rm{keV}}}}}}$$

For calcium mass and volume scores, a calibration factor c is needed. This factor was determined by measuring the attenuation of the CaHA and water calibration rods and using the following formula per keV level:

$${{{\rm{c}}}}=\frac{200{{{\rm{mg}}}}/{{{{\rm{cm}}}}}^{3}}{{{{{\rm{HU}}}}}_{{{{\rm{CaHA}}}}}-{{{{\rm{HU}}}}}_{{{{\rm{water}}}}}}$$

Agatston, volume and mass scores and image noise (standard deviation of HU values measured in a 1.5 cm^2^ region of interest within the water-equivalent insert) were analyzed. For noise analysis, a lower target was set at 20 HU for the small and medium phantom and at 23 HU for the large phantom to keep the radiation dose reasonable [22]. Additionally, an upper threshold at 30 HU for the small and medium phantom and at 35 HU for the large phantom was used to prevent false-positives [7].

Statistical analyses were performed using GraphPad Prism (Version 8.4.2; GraphPad). Continuous variables are reported as median with interquartile range (IQR), and categorical variables as absolute frequencies and proportions. The use of median with IQR down-weights outliers and may understate the impact of individual extremes. However, the absolute range (min – max) is additionally illustrated in the figures. *p* < 0.05 was considered statistically significant.

To assess interscan variability, the IQR of total Agatston scores from all five interscan variability examinations and phantom sizes was calculated. As the variability values represent comparative reproducibility measures across protocols, formal statistical testing was not applied.

In this part of the study (Part II), the optimized PCD-CT protocol from Part I was further refined by analyzing different VMI levels. Scores of this further adjusted protocol were compared to the optimized PCD-CT protocol from Part I, the standard PCD-CT protocol (120 kVp, 100% dose, thick slices), and the standard and proposed EID-CT protocols [7].

To differentiate the various protocols in a concise way, we refer to them as follows:

- Optimized PCD-I protocol from Part I: 120 kVp, 75% dose, 1.0 mm slices, IR 2
- Optimized PCD-II protocol from Part II, which is based on the optimized PCD-I protocol, but includes additional keV analyses
- Standard PCD protocol: 120 kVp, 100% dose, 3.0 mm slices
- Standard EID protocol: 120 kVp, 100% dose, 3.0 mm slices, filtered back projection
- Proposed EID protocol [7]: 100kVp, 75% dose, 1.0 or 1.25 mm slices, iterative reconstruction

Score variability was evaluated using the following formula: [7]

$$\left(-1+\frac{{{{{\rm{IQR}}}}}_{{{{\rm{new}}}}\; {{{\rm{protocol}}}}}}{{{{{\rm{IQR}}}}}_{{{{\rm{previous}}}}\; {{{\rm{protocols}}}}}}\right){{{\rm{x}}}}100 \%$$

Kruskal–Wallis test as well as Mann–Whitney U test with Bonferroni correction (*p*-value of 0.05/7 = 0.008) was used to assess differences between Agatston scores at various VMI levels using the current standard CAC threshold of 130 HU. Differences in detected calcifications between different VMI were compared using paired *t*-test. Mann–Whitney U test was used to analyze noise differences between the optimized PCD-II, the optimized PCD-I, as well as the proposed EID-CT protocol. Bonferroni correction was applied (*p*-value of 0.05/3 = 0.017).

## Results

### Adapted CAC threshold

**Fig. 1:**
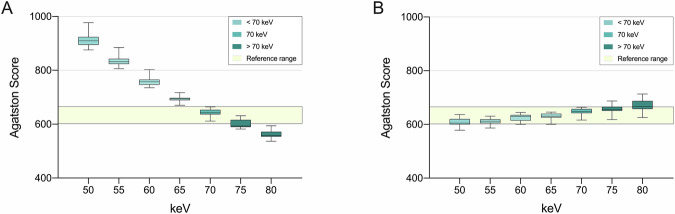
Total Agatston scores at different virtual monoenergetic image (VMI) levels using **A** the standard coronary artery calcium (CAC) threshold at 130 HU as well as **B** the adapted CAC thresholds, each exemplary for the optimized photon-counting detector (PCD)-I protocol (120 kVp, 75% radiation dose, 1.0 mm slice thickness, IR strength 2). The light-yellow range represents the interquartile range (IQR) of all Agatston scores from four different energy-integrating detector (EID)-CT scanners using the standard EID-CT protocol

The analysis of CAC scoring using the standard CAC threshold at 130 HU showed significant differences across all VMI levels (*p* < 0.001) with significantly increasing scores at lower keV (*p* < 0.001) and significantly decreasing scores at higher keV (*p* < 0.001) compared to the current standard VMI level at 70 keV. The corresponding Agatston scores are shown, exemplary for the protocol proposed in Part I, in Fig. 1. Correspondingly, measuring the phantom’s calcium calibration insert demonstrated increasing HU values at lower VMI levels and decreasing HU values at higher VMI levels. Based on the individual HU values, adapted CAC thresholds were determined for different VMI levels, as presented in Table 1. For further analyses, only adapted thresholds were used.

**Table 1:** Adapted CAC threshold for different VMI settings

VMI level	Mean HU_CaHA_	Adapted CAC threshold
50 keV	420.4	217
55 keV	356.8	187
60 keV	317.7	164
65 keV	280.2	144
70 keV	252.1	130
75 keV	230.2	119
80 keV	211.0	109

*CAC* coronary artery calcium, *CaHA* calcium hydroxyapatite, *HU* Hounsfield units, *VMI* virtual monoenergetic image

### Reproducibility of CAC quantification

**Fig. 2:**
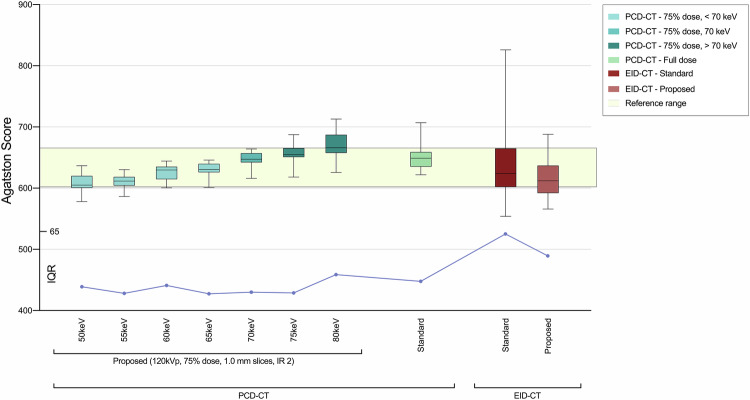
Total Agatston scores of the optimized photon-counting detector (PCD)-I protocol at different virtual monoenergetic image (VMI) levels, in comparison to Agatston scores obtained from the standard PCD protocol, as well as four state-of-the-art energy-integrating detector (EID)-CT scanners by using the standard and previously proposed EID protocols. Additionally, the blue graph illustrates the interquartile range (IQR) per protocol. The light-yellow range represents the IQR of all Agatston scores from those four EID-CT scanners using the standard EID-CT protocol

Figure 2 illustrates the Agatston score variability across different VMI levels acquired from the optimized PCD-II protocol, compared to the optimized PCD-I, the standard PCD, as well as standard and proposed EID-CT protocols.

Using the optimized PCD-I protocol at different VMI, the lowest Agatston score variability was achieved at 55, 65, and 75 keV with a decrease of 6%, 9%, and 4%, respectively, compared to 70 keV.

### Image noise and calcification detectability

**Fig. 3:**
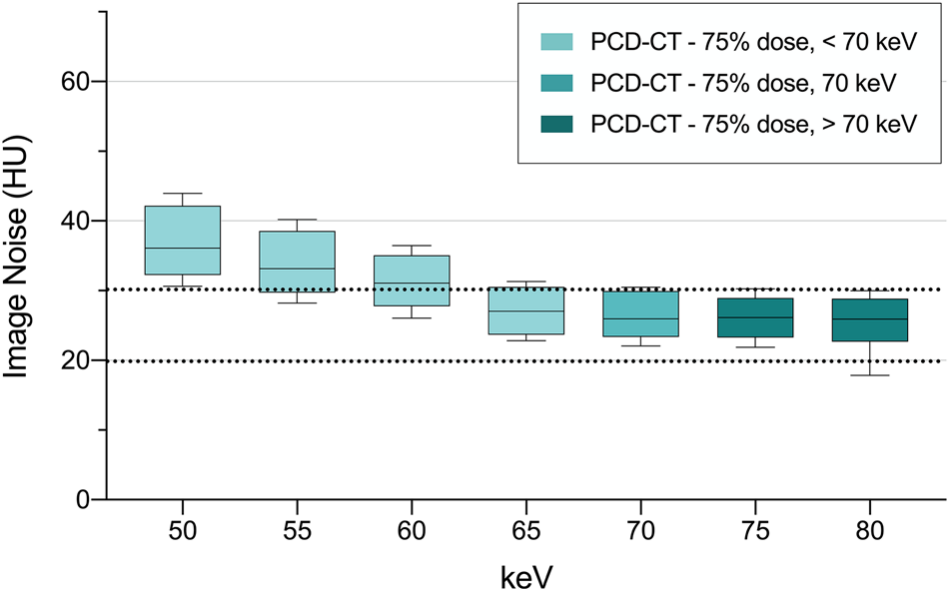
Image noise levels of the optimized photon-counting detector(PCD)-I protocol (120 kVp, 75% radiation dose, 1.0 mm slices, IR strength 2) at different virtual monoenergetic image (VMI) levels. The range between the dashed lines indicates the target range

Due to the higher risk of false-positives, protocols exceeding the upper noise threshold were automatically not further considered for protocol recommendation. Image noise was within the upper and lower threshold when using VMI levels higher than 60 keV. Corresponding image noise at 50 to 60 keV exceeded the upper threshold. Median image noise values are illustrated in Fig. 3.

The number of detected calcifications per VMI level is shown in Table 2. Using the optimized PCD-I protocol at 70 keV, 7.1 ± 0.8 of the nine calcifications were detected. For the optimized PCD-II protocol, the detectability of calcifications was significantly lower at 50 keV (*p* = 0.001), 55 keV (*p* = 0.004), and 75 keV (*p* = 0.04), and similar to 70 keV at 60, 65, and 80 keV (*p* = 0.08, *p* = 0.67, and *p* = 0.67).

**Table 2:** Number of detected calcifications using the protocol proposed in Part I (120 kVp, 75% radiation dose, 1.0 mm slices, IR strength 2) at different VMI settings

VMI level	Number of calcifications
50 keV	6.2 ± 0.4
55 keV	6.3 ± 0.5
60 keV	6.7 ± 0.5
65 keV	7.1 ± 0.6
70 keV	7.1 ± 0.8
75 keV	6.9 ± 0.8
80 keV	7.2 ± 0.7

*VMI* virtual monoenergetic image, *IR* iterative reconstruction

### Optimized PCD-II protocol—comparison to the optimized PCD-I, standard PCD as well as EID protocols

Based on the results mentioned above, a VMI level of 65 keV (in combination with 120 kVp, 75% radiation dose, 1.0 mm slices, IR 2) led to the lowest score variability, no exceedance of the upper image noise threshold, and the highest detectability of calcifications.

This VMI-optimized PCD-II protocol resulted in a reduced Agatston score variability by 9% compared to the optimized PCD-I protocol at 70 keV, by 43% compared to the standard PCD protocol, by 78% compared to the standard EID protocol, and by 69% compared to the previously proposed EID protocol. Comparing the optimized PCD-II protocol with the most comparable EID-CT scanner from the same vendor (SOMATOM Force, Siemens) showed a lower Agatston score variability by 33% compared to the standard EID protocol and by 23% compared to the previously proposed EID protocol. Corresponding changes of calcium volume and mass score variability are summarized in the Supplementary Results.

For the small, medium, and large phantom, image noise of the optimized PCD-II protocol at 65 keV was 23.7 (23.2–24.9), 27.1 (25.3–27.7), and 30.1 (30.0–30.3), respectively. Those values did not significantly differ from the corresponding image noise levels obtained with the optimized PCD-I protocol at 70 keV (*p* = 0.22, *p* = 0.19, and *p* = 0.10, respectively). Compared to image noise values obtained with the proposed EID protocol, corresponding values were similar for the small and medium phantom (*p* = 0.22 and *p* = 0.15) but significantly higher for the large phantom (30.1 [30.0–30.3] vs. 28.4 [27.0–28.8]; *p* = 0.008), which was below the upper threshold for the large phantom [7]. Although mostly no statistically significant differences were detected between the protocols, this does not imply complete equivalence. Instead, the findings indicate comparable performance within the limits of the present experimental setup.

The detectability of calcifications was 7.1 ± 0.6 using the optimized PCD-II protocol at 65 keV, 7.1 ± 0.8 using the optimized PCD-I protocol at 70 keV (see Part I), 6.0 ± 0.0 using the standard PCD protocol, 6.2 ± 0.4 using the standard EID protocol, and 7.0 ± 0.4 using the proposed EID protocol.

### Per-calcification analysis

With the optimized PCD-II protocol at 65 keV, median Agatston scores of 800 mg/cm^3^ CaHA decreased by 4% for the large- and 5% for the medium-sized calcifications compared to the optimized PCD-I protocol at 70 keV and by 12% and 16%, respectively, compared to the standard PCD protocol. Corresponding values of 400 mg/cm^3^ CaHA changed by −2% and −3% compared to the optimized PCD-I protocol at 70 keV and by 1% and 15% compared to the standard PCD protocol. Median Agatston scores of 200 mg/cm^3^ CaHA large- and medium-sized calcifications changed by −4% and −2% compared to the optimized PCD-I protocol at 70 keV and by 48% and 26% compared to the standard PCD protocol.

Calcification volumes and mass scores of the optimized PCD-II protocol were closer to the physical volume and mass of all calcifications than corresponding scores obtained using the standard PCD protocol (see Supplementary Results and Supplementary Figs. S1 and S2).

Using the optimized PCD-II protocol at 65 keV led to the following changes in score variability per calcification compared to the optimized PCD-I protocol at 70 keV (see Part I): Variability changed by 0.5%, 5.0%, and 7.0% for large-sized calcifications at 800 mg/cm^3^ CaHA, 400 mg/cm^3^ CaHA and 200 mg/cm^3^ CaHA, respectively, and by −1.4%, −43.9%, and −2.3% for medium-sized calcifications at 800 mg/cm^3^ CaHA, 400 mg/cm^3^ CaHA and 200 mg/cm^3^ CaHA, respectively.

**Fig. 4:**
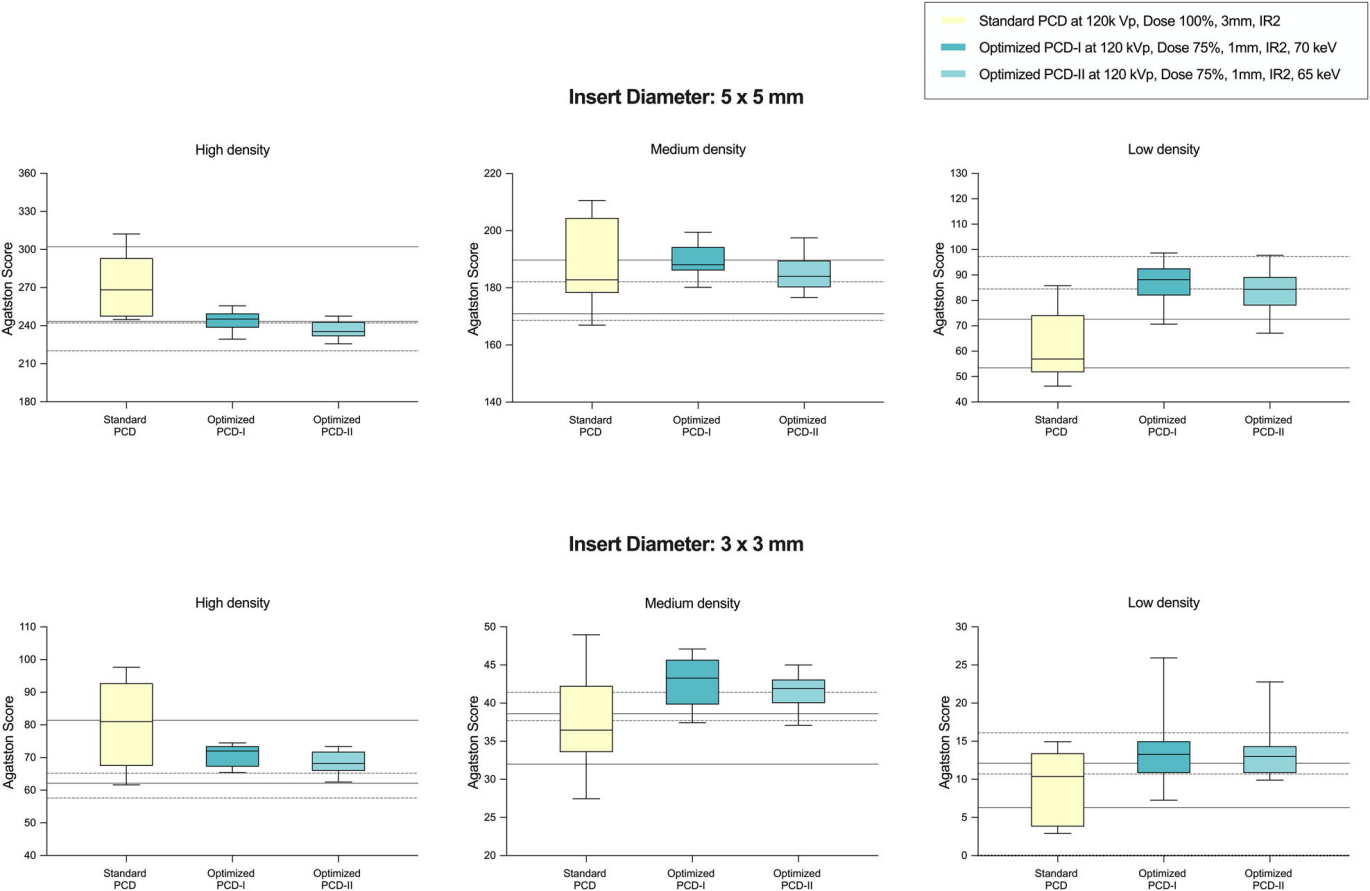
Per calcification analysis of Agatston scores using the standard, the optimized photon-counting detector (PCD)-I and PCD-II protocols. The continuous lines illustrate the interquartile range of the Agatston scores using the energy-integrating detector (EID)-CT standard protocol. The dashed lines show the interquartile range of the Agatston scores using the previously proposed EID-CT protocol. The illustration of the 1 mm diameter calcifications was omitted, as these calcifications have not been detectable with every protocol. Calcifications with 800 mg/m³ calcium hydroxyapatite (CaHA) are defined as “High density,” with 400 mg/m³ CaHA as “Medium density,” and with 200 mg/m³ CaHA as “Low density”. IR, iterative reconstruction

Compared to the standard PCD protocol, Agatston score variability decreased by −74%, −62%, and −51% for large-sized 800, 400, and 200 mg/cm^3^ CaHA, and by −81%, −59%, and −62% for medium-sized 800, 400, and 200 mg/cm^3^ CaHA calcifications, respectively. Corresponding changes compared to the standard EID protocol were −83%, −60%, −49% and −75%, −62%, −45%, compared to the proposed EID protocol −54%, −45%, −23% and −36%, −26%, −40%. The comparison of the Agatston score variabilities between the different protocols is illustrated in Fig. 4.

## Discussion

In Part I of this study, we proposed a dose-reduced PCD-CT protocol resulting in improved CAC score variability and calcification detection. Part II further investigated the reproducibility of PCD-CT-based CAC scoring at different VMI, including CAC threshold adjustments, and provided a dose-reduced protocol with further improved score variability. The main findings are: First, the lowest score variability was obtained at 55, 65, and 75 keV. Second, noise thresholds to prevent false-positives were not exceeded at ≥ 65 keV. Considering VMI ≥ 65 keV, the number of detected calcifications was only similar to the current standard (70 keV) when using 65 keV. Third, based on the results from Parts I and II, a protocol at 120 kVp, 75% radiation dose with thin slices, IR 2, and 65 keV was proposed, resulting in reduced score variability by 9% compared to the optimized PCD-I protocol at 70 keV, by 43% compared to the standard PCD protocol, by 78% compared to the standard EID protocol, and by 69% compared to the previously proposed EID protocol. The optimized PCD-II protocol also delivered a higher calcification detectability compared to previous protocols. Fourth, calcium volume and mass scores of the optimized PCD-II protocol were closer to the physical reference.

Recent studies have demonstrated the technical feasibility and diagnostic potential of PCD-CT-based VMI for cardiovascular applications [23, 24]. Different VMI levels influence CAC scoring [14, 25], with higher scores at lower keV [18, 26] and lower scores at higher keV [27] compared to the 70 keV standard. This is due to increased calcium attenuation at low keV, confirmed in this current study by higher HU values measured in the phantom’s calcium rod, resulting in higher calcium blooming [28]. In the present study, CAC scores at various VMI levels differed significantly between different keV when adhering to the current standard CAC threshold of 130 HU. Therefore, thresholds must be adjusted for each VMI to match results at 70 keV. For CAC scoring at different tube voltages, this has already been performed successfully by measuring mean calcium attenuation values [20, 21]. The current study indicates the transferability of this calculation to CAC scoring at different VMI with scores closer to the current standard of 70 keV.

Using these adapted CAC thresholds for different VMI in addition to the optimized PCD-I protocol further reduced score variability only at 55, 65, and 75 keV. This indicates a non-linear interaction between image contrast, noise, CAC threshold, and keV level in PCD-CT-based CAC scoring, which should be further investigated in future studies. The optimal VMI was selected based on noise and calcification detectability. It is known that decreasing keV levels increase noise [29–31]. This was also observed in the present study: Only VMI settings ≥ 65 keV did not exceed the noise threshold. Calcification detectability was only comparable to 70 keV when using 60, 65, and 80 keV, but significantly lower at other investigated keV levels. Due to higher contrast and proximity to calcium’s K-edge, lower keV levels are expected to improve detection rates, which has been observed in previous studies [11, 12, 15]. However, in the present study, the necessary CAC threshold adjustments counteract these density differences to keep scores comparable to currently used protocols. This, similar to the score variability analysis, results in a non-linear calcification detection rate based on the interplay between image contrast, noise, CAC threshold, and keV level.

Overall, the optimized PCD-II protocol (PCD-I protocol at 65 keV) showed the best results in score variability, image noise and calcification detectability. This further improved score variability compared to previous protocols. Future dynamic phantom and clinical studies are needed to validate these results. However, the improved score variability using this optimized PCD-II protocol indicates the potential of a more precise and consistent cardiovascular risk stratification and may support an improved individual clinical decision-making regarding preventive therapies for cardiovascular diseases.

While the optimized PCD-II protocol improved reproducibility under controlled conditions, achieving this in clinical follow-up examinations would probably require scanning the patients on the same system using identical acquisition and reconstruction parameters, which represents a general limitation of CAC follow-up imaging. Van Praagh et al [7] showed that the optimized protocol also reduced interscanner variability in EID-CT, suggesting a more comparable CAC scoring across different systems. In this context, the optimized PCD-CT protocol developed in this study has the potential to contribute to a more consistent longitudinal CAC assessment. This study has several limitations. The analysis was based on a static phantom with specific calcifications. Future studies should investigate the impact of motion and a wider range of calcification densities and sizes. In Part I of this study, all analyses were performed at 70 keV to reflect the current standard setting for CAC scoring. As Part II subsequently identified 65 keV as optimal, future studies should also re-evaluate the optimized PCD-CT protocol at 65 keV using the different acquisition and reconstruction parameters from Part I to further refine and validate our findings. We did not investigate the optimized PCD-CT protocol in patients. However, this in vitro analysis provides first systematic results and represents an essential basis for further in vivo studies. The study’s settings were specifically chosen to ensure comparability with the study by van Praagh et al [7] investigating a new CAC scoring protocol for EID-CT. This includes matching slice thickness and increment, as well as manually selecting radiation doses. However, these settings may not fully reflect clinical practice, where automatic exposure control systems are routinely applied. Additionally, oversampling techniques, such as using a lower increment than slice thickness, could further reduce score variability. Furthermore, this study was conducted using a single-vendor PCD-CT system. Therefore, the results may not be directly transferable to systems from other manufacturers, and reproducing these results in clinical follow-up would require using the same scanner and protocol. To ensure clinical applicability, future studies should investigate the optimized PCD-CT protocol under real-world clinical conditions as well as using other PCD-CT systems.

In conclusion, this study indicates that a PCD-CT protocol at 120 kVp, 75% radiation dose with thin slices, IR2 and 65 keV significantly improves CAC score variability and calcification detectability compared to the current standard PCD-CT protocol, as well as the standard and proposed EID-CT protocols, supporting more precise and consistent cardiovascular risk stratification with CAC scoring.

## Supplementary information

ELECTRONIC SUPPLEMENTARY MATERIAL

## Abbreviations

CAC: Coronary artery calcium
CaHA: Calcium hydroxyapatite
EID: Energy-integrating detector
HU: Hounsfield unit
IQR: Interquartile range
IR: Iterative reconstruction
keV: Kilo-electron volt
PCD: Photon-counting detector
VMI: Virtual monoenergetic image
