# Supplementary material for: Coronary artery calcium scoring: expanding the new standard by photon-counting detector CT Part II: Impact of virtual monoenergetic image reconstructions with adjusted calcium scoring thresholds
Source: Eur Radiol. 2026 Feb 19;36(7):5557–66. doi: 10.1007/s00330-026-12356-3 (PMC13282229; doi:10.1007/s00330-026-12356-3)
Supplement: Supplementary file 1 — ELECTRONIC SUPPLEMENTARY MATERIAL [file 330_2026_12356_MOESM1_ESM.pdf]

Coronary Artery Calcium Scoring: Expanding the New Standard by Photon-Counting Detector CT Part II: Impact of Virtual Monoenergetic Image Reconstructions with Adjusted Calcium Scoring Thresholds

ELECTRONIC SUPPLEMENTARY MATERIAL

Supplementary Results

Reproducibility of CAC quantification

Overall, calcium volume and mass score analyses showed similar trends as Agatston scores with a reduction of score variability when using 65keV compared to 70keV.

Using the optimized PCD-I-protocol at different VMI, in comparison with 70keV, calcium volume score variability was only lower at 65keV, where it was reduced by 9%.

Corresponding calcium mass variabilities were improved when using the optimized PCD-I protocol at VMI below 70keV with a reduction of score variability by -4%, -13%, -8%, and -11% at 50, 55, 60, and 65keV, respectively. Compared to the optimized PCD-I protocol at 70keV, mass score variability increased by 9% when using 75keV and by 35% when using 80keV.

Optimized PCD-II Protocol – Comparison to the optimized PCD-I, standard PCD and EID protocols

The VMI-optimized PCD-II protocol at 65keV resulted in a reduction of calcium volume variability by 9% compared to the optimized PCD-I protocol, by 49% compared to the standard PCD protocol, by 91% compared tot the standard EID protocol and by 80% compared to the proposed EID protocol.

Using the PCD-II protocol at 65keV changed mass score variability by -11% compared to the optimized PCD-I protocol, by 9% compared to the standard PCD protocol, by -71% compared to the standard EID and by -68% compared to the proposed EID protocol.

Per-Calcification Analysis

Compared to the standard PCD protocol, median calcium volume scores changed by -18% and -22% for the large- and medium-sized 800 mg/cm^3 CaHa calcifications, by -14% and -4% for the large- and medium-sized 400 mg/cm^3 CaHa calcifications, and by 5% and 73% for the large- and medium-sized 200 mg/cm^3 CaHa calcifications. Compared to the optimized PCD-I protocol, calcium volume score variability changed by -36%, -16%, and 126% for large-sized 800, 400, and 200 mg/cm^3 CaHa, and by -30%, -18%, and -8% for medium-sized 800, 400, and 200 mg/cm^3 CaHa calcifications. Respective changes were -88%, 26%, -2%, -88%, -7%, and -14% compared to the standard PCD protocol, -95%, -85%, -65%, -93%, -70% and -12% compared to the standard EID protocol and -84%, -73%, -46%, -88%, 7%, and -17% compared to the proposed EID protocol.

Using the VMI-adjusted PCD-II compared to the standard PCD protocol, median calcium mass scores changed by -2% and 0% for the 800 mg/cm^3 CaHa large- and medium-sized calcifications, by -3% and -2% for the 400 mg/cm^3 CaHa large- and medium-sized calcifications, and by -2% and -3% for the 200 mg/cm^3 CaHa large- and medium-sized calcifications, respectively. Mass score variability changed by 53%, 30%, and 22% for large-sized 800, 400, and 200 mg/cm^3 CaHa, and by -34%, -8%, and -14% for medium-sized 800, 400, and 200 mg/cm^3 CaHa, each compared to the optimized PCD-I protocol. Corresponding changes were -38%, 173%, -53%, -44%, 16%, -42% compared to the standard PCD protocol, -62%, -42%, -69%, -74%, -52%, -24% compared to the standard EID-CT protocol and -61%, -16%, -50%, -76%, -4%, -35% compared to the proposed EID protocol.

Supplementary Figures

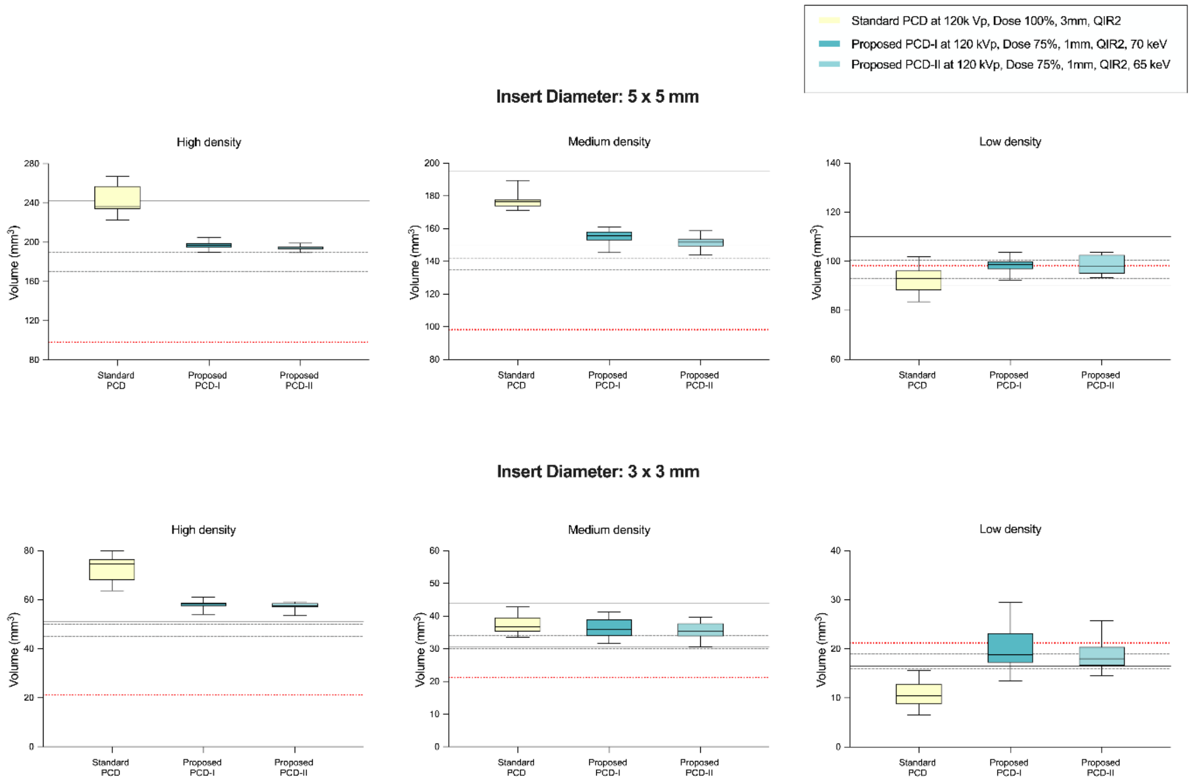

Figure S1: Per calcification analysis of calcium volume scores using the standard, the optimized photon-counting detector (PCD)-I and PCD-II protocols. The continuous lines illustrate the interquartile range of the Agatston scores using the energy-integrating detector (EID)-CT standard protocol. The dashed lines show the interquartile range of the Agatston scores using the previously proposed EID-CT protocol. The illustration of the 1 mm diameter calcifications was omitted, as these calcifications have not been detectable with every protocol.

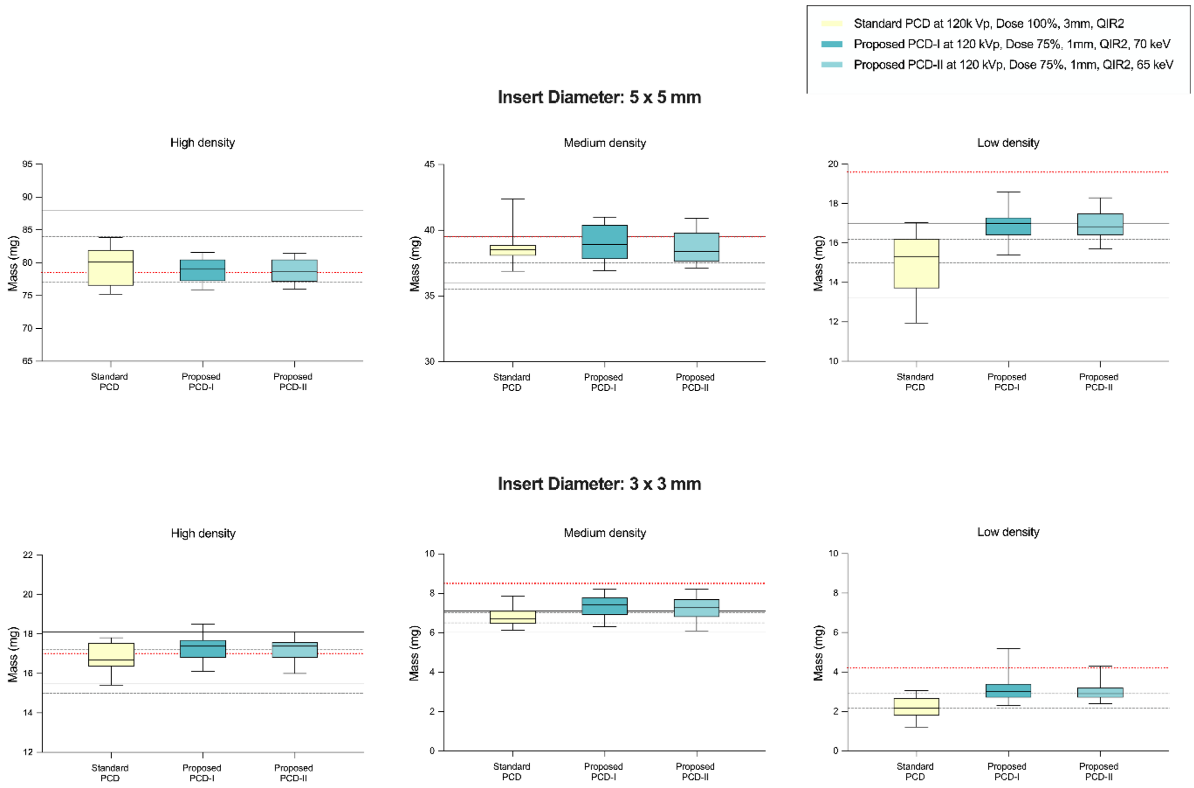

Figure S2: Per calcification analysis of calcium mass scores using the standard, the optimized photon-counting detector (PCD)-I and PCD-II protocols. The continuous lines illustrate the interquartile range of the Agatston scores using the energy-integrating detector (EID)-CT standard protocol. The dashed lines show the interquartile range of the Agatston scores using the previously proposed EID-CT protocol. The illustration of the 1 mm diameter calcifications was omitted, as these calcifications have not been detectable with every protocol.
